# Wandering Drunkards Walk after Fibonacci Rabbits: How the Presence of Shared Market Opinions Modifies the Outcome of Uncertainty

**DOI:** 10.3390/e26080686

**Published:** 2024-08-13

**Authors:** Nicolas Maloumian

**Affiliations:** R&D, Primum Tempus, 66 Prescot Street, London E1 8NN, UK; nicolas.maloumian@primumtempus.com; Tel.: +33-6-0885-5320

**Keywords:** value, opinions, price, Fibonacci ratio, G10, G12, G17, E37

## Abstract

Shared market opinions and beliefs by market participants generate a set of constraints that mediate information through a not-so-unstable system of expected target prices. Price trajectories, within these sets of constraints, confirm or disprove the likelihood of participant expectations and cannot, de facto, be considered permutable, as literature has shown, since their inner structure is dynamically affected by their own progress, suggesting per se the presence of both heat and cycles. This study described and discussed how trajectories are built using different alphabets and suggests that prices follow an ergodic course within structurally similar tessellation classes. It is reported that the courses of price moves are self-similar due to their a priori structure, and they do not need to be complete in order to create the conditions, in resembling conditions, for the appearance of the well-known and commonly used Fibonacci ratios between price trajectories. To date, financial models and engineering are mostly based on the mathematics of randomness. If these theoretical findings need empirical validation, such a potential infrastructure of ratios would suggest the possibility for a superstructure to exist, in other words, the emergence of exploitable patterns.

## 1. Introduction

Market participants define their expectations based on beliefs that are supposed to be valid and shared market opinions built on models understood as justified. Most of the time, these shared beliefs and opinions result from identical causal grounds and from the same forms of analysis methods. Their existence is based upon common and articulated reasoning and, as such, tends to group into a limited set of expectations. Investor sentiments result from these articulated, shared, and henceforth, valid opinions and beliefs, which affect financial market prices [1,2]. Moreover, a set of shared forecasts exerts a structural constraint on price by mapping the time-price plane. This set is, by nature, more stable than the information received as long as market participants are varied in their purchasing or selling power and have differing time horizons (different groups of expectations) [3]. In other words, this set of target prices stands as an intermediary between information, which includes price and price change [3].

As such, the set, consisting of information, value and price, is a complex system with feedback loops. Non-negligible effects emerge from the progressive adjustments of price within the structure of expectations. The uncertainty of a market price is subjected to a physical system led and constrained by goals. It is difficult to understand how prices progress by analysing data with statistical models that do not consider the web of constraints exerted on prices by investor expectations. This could be seen as a fundamental error since these constraints disappear as price history is established. There is a metaphysical component to price construction that is not taken into account by statistical models and was the primary omission in Bachelier’s seminal work, which propagates to this day. Integrating these constraints into the understanding of the structure of price histories would mean uncovering properties of self-similarity in the progressions and thus represents a change of paradigm [4].

Complexity should be considered more in economic theory and market finance research since it may have large and long-term epistemic implications. As with most true revolutions, complexity has come in by a little door in the backstage of official or normal science, as Thomas Kuhn coined it. In the classical world of economic sciences and market finance, research has become, from the early days of the 20th century to today, a mathematised field subjected to models borrowed from various disciplines, generally applied mathematics and physics, in which both worlds have brought their contributions of equations and models, and also its share of needed and demanding hypotheses to adapt and sometimes force *reality* to apply the models in order to solve complicated problems with generally complicated (differential) equations [5]. The nature of the changes that complexity may bring could be devastating to both models and hypotheses because sciences based on classical epistemic paradigms may lose their foundational solidity without any consideration for the effort to maintain the hypotheses or any validity regarding the worth of empirical and statistical validation. In other words, complexity radically modifies our appraisal of the notion of causality.

It is not only that complex systems may show unforecastable behaviours even given the most conservative hypotheses, but also that these unforecastable behaviours may remain invisible considering the use of large datasets, as the well-documented and virtual Langton’s ant dynamics has shown. This is why complex systems can only be understood by experiencing, more than experimenting, the system in which the ‘experience’ is radically different to analysing data; only there could the term empirical substantiation have a meaning.

In the financial markets, the computational irreducibility emerging from the complex relationship between shared market opinions, price, value and information cannot be neglected since it has an impact on any hypotheses regarding the supposed randomness of price behaviours and all the epistemic bias that hypotheses may carry and spread [6]. The link between price and value, price being the measure of value, cannot be that natural. There is a revolution of the mind that has not yet taken place, starting with the vocabulary and the pre-supposed oriented chain of causes and effects. To illustrate this, the ‘irrational exuberance’ of Alan Greenspan could be understood as a sign of epistemic subjectivity or ignorance of a phenomenon.

Over the years, there have been many signs showing that bullish and bearish market sequences have been ‘experienced’ by market participants to be structured by Fibonacci ratios, which are present in other complex systems, including in the physical and biological world of botany and zoology [7,8,9,10]. Literature on the empirical evidence of the presence and use of Fibonacci ratios in the markets is abundant and available on all charting software [11,12,13,14]. However, to our knowledge, there is no theoretical evidence of them appearing. The objective of this study was to theoretically describe the rationale for the emergence of Fibonacci ratios without numerical evidence and not based on statistical data.

## 2. Methods

Obtaining a clean and accurate dataset from an asset traded with significant volume is a challenge. This study applied a multidisciplinary approach using tessellation classes, Fibonacci tiling properties and homomorphism of the tiling system to cover the study objectives. To theoretically address how price formation relates to Fibonacci ratios, the task was to show how the probabilistic distribution that could govern trades can be reduced to a path-dependent Fibonacci one, which naturally weighs on price change. The starting point was an aporia in Bachelier’s seminal thesis, which led to the clarification of the difference between pathwise and path dependency dynamics [15]. If the constituents of a marketplace are various and numerous (such as type of market, volume, arbitrage, liquidity providers, hedging and access costs) and structure market price evolution, it would ultimately be the struggle between sellers and buyers that moulds market price paths. A market is bullish or bearish as the buyers or the sellers, respectively, are in control and exert their control through their most shared opinions and beliefs regarding value, which, in turn, shape the space of possible prices.

Understanding the practical impact of this context is not an evident task since price history, a concrete object, is a result, and one based on circumstances which gradually disappear along the production of this result. This context is the network of the expected prices (the expression in numerical terms of the different possible expected values) that existed at the time the market moved up or down. During the processing of price, this context, or receptacle, is stable as long as information, which includes price, does not dramatically change expectations, for instance, those based on longer time horizons [3,16].

The next step was to consider a simple theoretical alphabet of four letters {α, u, d, H}, where α is the first price, ‘u’ means a tick up, ‘d’ a tick down and ‘H’ an unchanged price when compared to the former price. A ‘word’ written on this four-letter alphabet can describe any market phase, for instance, in considering the word ‘αuuHuHdHduddHu…ddHuHuu’. Words can be used to describe sequences of up-market phases or down-market phases, as in ‘αuuHuHuHuuu’ or in ‘αdHddHddd’. Such sequences within words are built on even more simple alphabets of three letters, {α, u, H} and {α, d, H}. The adaptation of prices to the locally most shared expectation of value is structured in a particular way, an inner architecture of market phases which is well described by words built on these two alphabets. By grouping these sequences into sets of identical lengths, called sets of tiles, it is possible to depict all the different possibilities of what can be considered a market phase, from bottom to top or from top to bottom, as human eyes see them.

Market phases tend to be self-similar as the way they are built is a self-similar progression caused by the constraints in which they evolve. This study also aimed to show how these prices’ bullish or bearish sequences naturally tend to relate to one another by a multiple of Fibonacci ratios using the properties of Fibonacci tiling systems since the overall structure of each market price phase is identically homomorphic, and since the causes of market moves (new information, fear, greed) are self-similar in regards of the context in which price moves occur.

## 3. Discussion

Given that a sufficient trading volume is present, prices result from a process which can be identified as a ‘vote’ [17]. This vote is based on available information, expressing how market participants interpret current prices relative to what their views of ‘value’ are, which was reflected in the thesis of Louis Bachelier, who compared price change to the result of a vote in his theory of speculation [15].

### 3.1. Bachelier and Methods of Vote Counting

The French national archives kept a record of Henri Poincaré’s official report on Bachelier’s thesis [18]. Poincaré detailed Bachelier’s methods, for example, on the mobilisation of the analytical theory of heat propagation to explain how probabilities ‘radiate’, using Bachelier’s terminology. He explained how the difficulty in linking the probability for a price to reach a certain level before a specific date with a ‘ballot count’ was overcome using an analogy with a theorem regarding vote counting that the mathematician Désiré André (1840–1917) demonstrated in 1887 with

‘If A and B are two candidates and A was getting *n* votes and B was getting *m* votes at the end of the voting process, with *n* > *m*, the probability P_A_ that A is always ahead of B at any time during the vote would be (*n* − *m*)/(*m* + *n*)’ [18,19].

Despite the novelty of his proof, Bachelier’s analysis of price behaviour only considered the values *after* the voting outcomes described the possible paths (the *m* and *n* from his thesis). It was a retrospective pathwise consideration, given an a posteriori result of the final repartition as clearly shown in his statement ‘at the end of the voting process’ where he did consider neither the process in its dynamic progression nor the constraints that could have been present [15,18,19]. 

In the financial markets, the ‘ballot box’ is transparent: voters can observe how close the results of the voting process are, which is an immediate consequence since the ‘votes’ and their repartition both in and against the order book may change at each lodging (in other words, at each price). In the case of the evolution of price, it therefore resembles a process with a negative feedback mechanism, as the price is close to the expectations. This control process changes the voting intentions in the function of its successive results and of shared market opinions.

### 3.2. Pathwise Is Not Path Dependency

Bachelier certainly had a pathwise approach to price evolution understood as a stochastic process, however, he may have ignored path dependency effects [4]. The cost here would have been the unreported presence of non-commutativity, the failure to see that different meanings may be attributed to a different price ‘history’ and, therefore, needs the consideration of how the voting process could be significantly impacted. Ignoring non-commutativity was, therefore, contradictory to the reality that prices could even reflect the effect of information on value, in other words, may confirm or refute shared opinions [20,21]. On the contrary, contemplating the effects of non-commutativity in the way prices are posted restores meaning to price trajectories. Be it in geometry or algebra, these effects lead to the appearance of heat and cycles [22]. It is worth noting that the behaviour of complex systems, such as in the financial markets, in any case, should not be considered through statistical analysis [23].

### 3.3. The Price Approach

Since the behaviour of a complex system can only be observed through experimentation, price movements are more important than price changes. As such, a distinction must be made between a chain of prices and a chain of trades.

**Figure 1:**
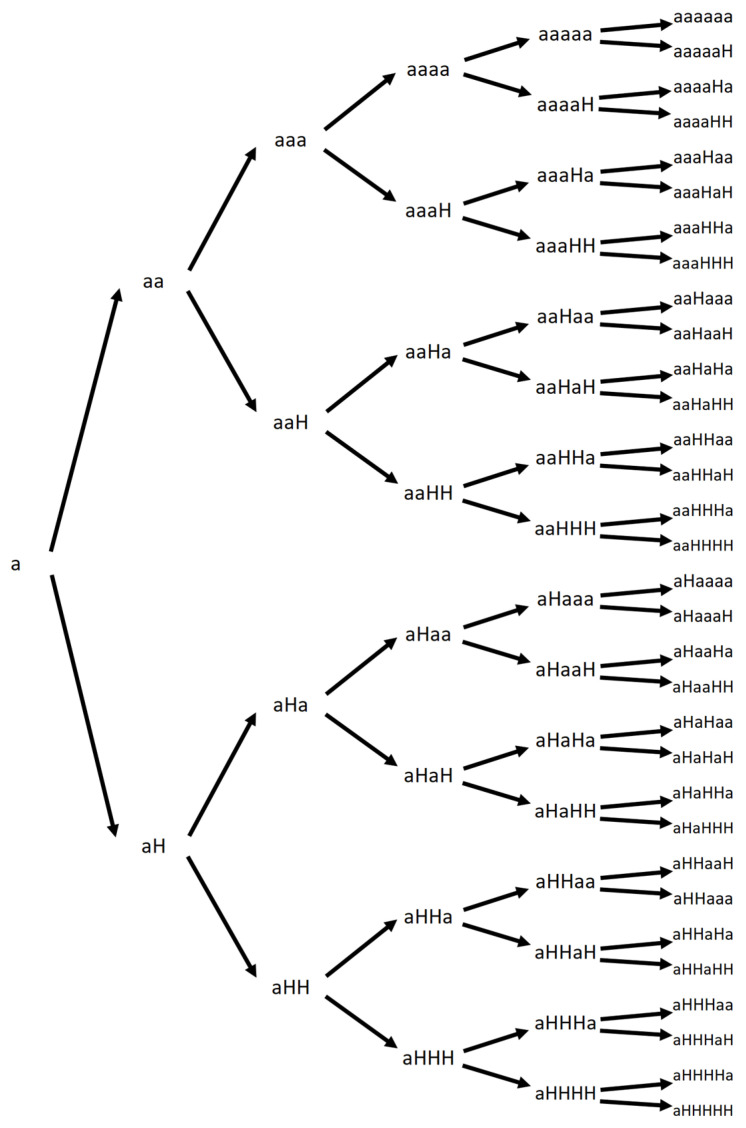
Different possible combinations after six trades, ‘a’ marking any price change, ‘H’ marking no change.

Let O be a set of m trades. O can be written [X_1_, X_2_, X_3_, …, X_m_], where X_i_ contains the trade price and trade size. As such, let P(X_i_) be the price of the trade Xi and S(X_i_) be the size of the trade X_i_. Let Q be the *n*-price chain; Q can be written using the symbols a_i_ [a_1_, a_2_, a_3_, …, a_n_] where a_i_ is a price that differs from its predecessor a_i−1_ and H standing for the h of ‘unchanged’ in case the price a_i+1_ is the same than a_i_. Each element of the *n*-price chain, be it a_i_ or H, has, as trades do, a price component and a size component -volume-, namely, P(a_i_) or P(H) and S(a_i_) or S(H). Price chains are random chains of price movements; for instance, after six trades, the possibilities are shown in Figure 1.

By convention, the first trade would be considered to be different from its predecessor; hence, none of the *n*-trade chains initiated by the letter H should be counted. 

Another grammar of prices can be chosen to represent market moves. Considering the following rules of composition, any price move can be written as a *n*-letter chain:

If P(X_1_) ≠ P(X_0_), Q = [] becomes Q = [a_1_], for the first price. (1)

If P(X_i+1_) ≠ P(X_i_), Q = [… a_i_ | H] becomes Q = [… a_i_ a_i+1_] or Q = [… H a_i+1_], (2)

If P(X_i+1_) = P(X_i_), Q = [… a_i_] becomes Q = [… a_i_ H] and, (3)

If P(X_i+1_) = P(X_i_) and P(X_i_) = P(X_i−1_), Q = […. a_i−1_ H] and remains identical since HH = H, (4)

where the symbol ‘|’ is ‘or’.

**Figure 2:**
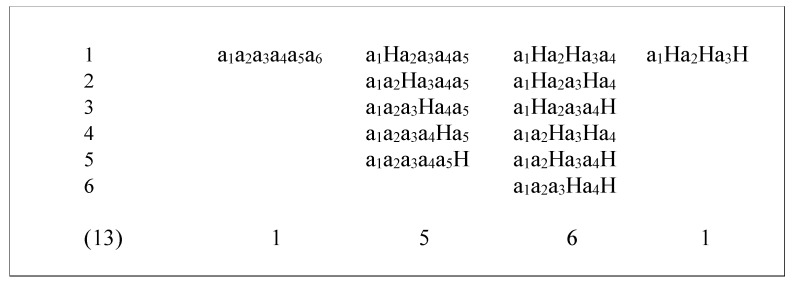
Different possible combinations posting a six-letter chain.

In the last configuration, the size component of H becomes the sum of S(H) and S(X_i+1_). Stationary or unchanged price is only signalled once: when considering an *n*-letter chain only, the elements showing two or more following H’s have to be removed. For instance, the chain a_1_a_2_HHHH is equivalent to the chain a_1_a_2_H, which should be considered as being a three-letter chain since there are only three letters written, not six. Considering a six-letter chain following the aforementioned rules will result in 13 possibilities for a six-letter chain, as shown in Figure 2.

T_P_ is defined as a function that depicts a *n*-letter chain:

(5) $$T_{P}\;(n)=C_{n}^{0}+C_{n-1}^{1}+C_{n-2}^{2}+\ldots +C_{n/2}^{n/2}$$

with *n*/2 terms, with *n* even.

(6) $$T_{P}\;(n)=C_{n}^{0}+C_{n-1}^{1}+C_{n-2}^{2}\;+\ldots +C_{(n+1)/2}^{(n-1)/2}$$

with (*n* + 1)/2 terms, with *n* odd. In both cases,

T_P_ (*n*) = *F_n_* (7)

where *n* is the number of letters used, $C_{n}^{p}$ is the combination of *p* elements among *n*, without order or repetition, *F_n_* the symbol of the Fibonacci sequence *F*_0_ = 1, *F*_1_ = 1, *F*_2_ = 2,…, *F_n_* = *F_n_*_−1_ + *F_n_*_−2_ implying respectively the following probability 1 = α_0_ + α_1_ + α_2_ + … + α_n/2_ and 1 = α_0_ + α_1_ + α_2_ + … + α_(n−1)/2_, where, for an even *n* and an odd *n*, and *n ≥* 5 (given the notation),

(8) $$\alpha_{0}=\frac{C_{n}^{0}}{F_{n}},\;\alpha_{1}=\frac{C_{n-1}^{1}}{F_{n}},\;\alpha_{2}=\frac{C_{n-2}^{2}}{F_{n}},\;\ldots ,\;\alpha_{n/2}=\frac{C_{n/2}^{n/2}}{F_{n}}$$

(9) $$\alpha_{0}=\frac{C_{n}^{0}}{F_{n}},\;\alpha_{1}=\frac{C_{n-1}^{1}}{F_{n}},\;\alpha_{2}=\frac{C_{n-2}^{2}}{F_{n}},\;\ldots ,\;\alpha_{(n-1)/2}=\frac{C_{(n+1)/2}^{(n-1)/2}}{F_{n}}$$

Taking into consideration that a market jump (or price gap) within a chain of price a_1_ a_2_|H a_3_|H … a_p_ a_p+1_ … a_n_|H, for instance, here between ap and ap + 1 can be written a_1_ a_2_|H a_3_|H … a_p_a_p+1_a_p+2_a_p+3_, a_p+j_ a_p+j+1_ … a_n+j_|H, considering that a market jump is just the posting of price with no volume attached, any price history can be described, with a minimum loss of information regarding the number of trades and the size of each trade during unchanged price phases. Additionally, if H is indexed and the list of volumes of each of the trades is kept for each indexed H, then at this stage, at least, the representation is neutral: any market history can be depicted by the representation and vice versa. Hence, the generally considered Pascal triangle’s probabilistic distribution that is generally used to depict trades can be reduced to a Fibonacci probabilistic distribution (one that governs actual price changes).

### 3.4. Alphabet and Words Describing Market Moves

In order to use the Fibonacci probabilistic distribution and observe how it describes the reality of market price moves, a rewriting of the chain of prices must be performed. The first step is, as aforementioned, the alphabet **A** = {a_1_, a_i_, H} to express any sequence of *n*-letter chain. Any sequence S would start with a beginning price a_1_ and thereafter would be composed of a_i_ and H in such a way that each H appearing in the sequence is either framed by an anterior a_i_ and a posterior a_i+1_ except for the last letter of the word which can be either an a_i_ or an H given its anterior is an a_i_.

Let **A’** = {α, U_i_, D_j_, H} be another alphabet, and **S** be a sequence of market prices composed of the alphabet **A**. Considering a function f: **A** to **A’** such that f transforms **S** on f(**S**), the transformations would be as follows:

(i) The first price is noted a;
(ii) Any a_i_ superior to its predecessor would become U_i_;
(iii) Any a_i_ inferior to its predecessor would become D_i_;
(iv) Any H would remain a H.

Hence, **S** = (a_1_a_2_Ha_3_Ha_4_H…a_n_) could be written as **S**’ = (α U_1_|D_1_ H U_2_|D_2_ H U_3_|D_3_ H…U_n_|D_n_) on **A’**, where | designates the ‘or’. 

Considering finally **A_+_’** = {α, u_i_, H} and **A_−_’**= {ω, d_j_, H}, two alphabets to compose **s^+^** and **s^−^** as positive and negative respectively price sequences where α, or ω, is, by convention, the first price of the respective chains, and where u_i_—resp. d_i_—are no more expressing the price U_i_—resp. D_i_—but the increase—resp. the decrease—in unit points, relative to the former price increase—resp. decrease—i.e., the difference U_i_ − U_i-1_—resp. the difference D_i_ − D_i−1_. 

Any *n*-letter chain **S** on **A**, and then on **A’** can therefore be written using **A_+_’** and **A_−_’** as a concatenation of **s**_i_^+^ and **s**_j_^−^, knowing that by convention, each last price u_n_ of each s_i_^+^ is the first ω price of each s_i_^−^ and resp. that each last price d_n_ of s_i_^−^ is the first α price of each s_i_^+^.

Lastly, the height of the sequence would be written as h(**S**) and would represent the number of unit prices gained or lost from the first price. To give an example, let us imagine a simple sequence of price Q, such as

**Q** = {1.0840, 1.0855, 1.0855, 1.0859, 1.0844, 1.0851, 1.0858, 1.0862, 1.0866, 1.0860, 1.0872, 1.0874}

**S**_{A}_ = {1.0840, 1.0855, H, 1.0859, 1.0844, 1.0851, 1.0858, 1.0862, 1.0866, 1.0860, 1.0872, 1.0874},

And S is a chain of price going up, as α_1_ = 1.0840 and the last price is 1.0874, with h(**S**) = 0.0034. Written with the alphabet A’, this chain becomes:

**S**_{A’}_ = {a_1_, U_1_, H, U_2_, D_1_, U_3_, U_4_, U_5_, U_6_, D_2_, U_7_, U_8_}, where each letter is a price of Q and

**S**_{A’}_ = {a_1_, u_1_…u_15_ H u_16_…u_19_; w_1_, d_1_…d_15_; a_2_, u_20_…u_41_; w_2_, d_16_…d_21_; a_3_, u_42_…u_55_},

where each letter u_i_ and d_i_ are, respectively, 0.0001 and -0.0001, and **S**_{A’}_ can be written as

**S**_{A’}_ = {{a_1_, u_1_…u_15_ H, u_16_…u_19_}, {w_1_, d_1_…d_15_}, {a_2_, u_20_ …u_41_}, {w_2_, d_16_…d_21_}, {a_3_, u_42_…u_55_}},

that can be written as **S**_{A’}_ = {**s**_1_^+^, **s**_1_^−^, **s**_2_^+^, **s**_2_^−^, **s**_3_^+^} and where α_1_ = 1.0840, ω_1_ = 1.0859, α_2_ = 1.0844, ω_2_ = 1.0866, α_3_ = 1.0860, and where **s**_1_^+^ **s**_2_^+^ and **s**_3_^+^ are written using **A_+_’** while **s**_1_^−^ and **s**_2_^−^ are written using **A_−_’**.

From there, another operation can take place, which consists of observing how prices, in this sequence **S**_{A’}_, go from 1.0840 to 1.0874 in a bullish sequence. As such, observing the ‘loops’ prices are making, from 1.0859 to 1.0844 and back to 1.0859, and from 1.0866 to 1.0860 and back to 1.0866, this word can be written, using only one alphabet, **A_+_’**, as

**Figure 3:**
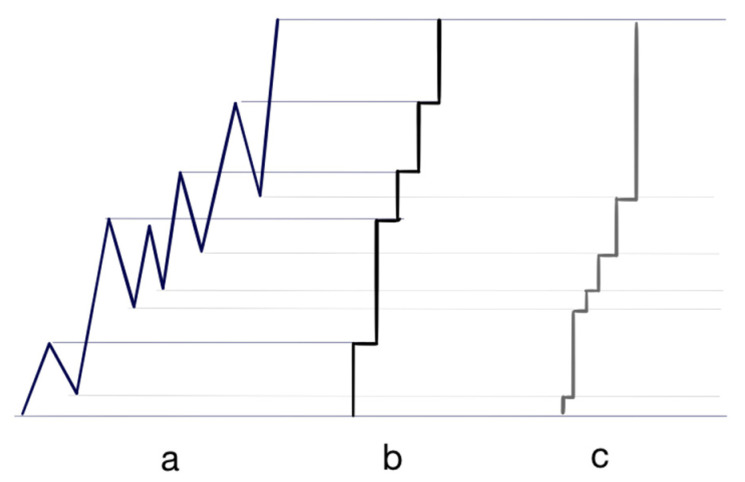
Different ways to see the same rise in price from a bottom to a top as depicted in ‘a’, with ‘b’ presenting an l-composition (l standing for left) of ‘a’, and with ‘c’ presenting an r-composition (r standing for right) of ‘a’. On both ‘b’ and ‘c’, each horizontal line is an ‘H’.

**S**_{A+’}_ = {a_1_, u_1_ … u_15_, H_1_, u_16_ … u_19_, H_2_, u_35_ … u_41_, H_3_, u_47_ … u_55_},

Hence, a new equivalent chain is obtained,

**S**_{A+’}_ = {a_1_, **uuuuuuuuuuuuuuu H**_1_**uuuu H**_2_**uuuuuuu H**_3_**uuuuuuuu**},

H_1_ is just provided here as an example of a price traded twice (1.0855) in this example, while H_2_ (1.0859) and H_3_ (1.0866) are two ‘buckles’ of the market. Taking now another example with the sequence described in Figure 3a, the corresponding sequence **S** can be seen as a sequence of six moves up and five moves down **s**_1_^+^, **s**_1_^−^, **s**_2_^+^, **s**_2_^−^, **s**_3_^+^, **s**_3_^−^, **s**_4_^+^, **s**_4_^−^, **s**_5_^+^, **s**_5_^−^ and **s**_6_^+^.

Figure 3b shows how all negative-signed sequences would be replaced by H to zoom out a positive-signed sequence. H are represented by the horizontal lines with, literally, a point of view from the left perspective. In this case, **S** becomes five different parts: **s**_1_^+^, **s**_2_^+^-{**s**_2_^+^∩**s**_1_^+^}, **s**_4_^+^-{**s**_4_^+^∩**s**_2_^+^}, **s**_5_^+^-{**s**_5_^+^∩**s**_4_^+^}, **s**_6_^+^-{**s**_6_^+^∩**s**_5_^+^} and is called a *l*-composition where *l* stands for left. Respectively, in an *r*-composition (*r* standing for right), there would be a union or the addition of **s**_i_^+^-{**s**_i_^+^∩**s**_i_^−^}, i evolving from one to six, knowing that the **s**_6_^−^ is empty (Figure 3c). Using the l-composition and r-composition provides two different sets to which the tiling structure can be applied to the same move and gives an additional chance to find a better fitting using a class of tiles of an *n*-letter chain. Both compositions create a different writing of the same sequence. For instance, in the previous composition,

Q = {1.0840, 1.0855, 1.0855, 1.0859, 1.0844, 1.0851, 1.0858, 1.0862, 1.0866, 1.0860, 1.0872, 1.0874}

was giving

**S**_({A+’}, Left)_ = {a_1_, **uuuuuuuuuuuuuuu H**_1_**uuuu H**_2_**uuuuuuu H**_3_**uuuuuuuu**}

where α_1_ = 1.0840, H_1_ = 1.0855, H_2_ = 1.0859, H_3_ =1.0866, in the *l*-composition, while, reading the chain from the right to the left, the **S**_{A+’}_ would have been described, in the *r*-composition, by

**S**_({A+’}, Right)_ = {a_1_′, **uuuu H_1_ uuuuuuuuuuuuuuuu H_2_ uuuuuuuuuuuuuuu**},

where α_1_′ = 1.0840 and where H_1_ =1.0844 and H_2_ = 1.0860. These two chains represent the same sequence, so they are two possible ways of describing the upward movement. This is an important point for the congruence of any price chain with the theoretical model, as it is possible to have two ways of writing it a posteriori.

### 3.5. Self-Similarities within the Compositions of Market Price Sequences

Each market sequence, as defined in Figure 3b,c, can physically be segmented or be made the object of a tiling by its most adequate, even though not necessarily exact, *n*-letter chain structure. The symbolism chosen allows for a description of any market increase or decrease as if it were a subset of the set of Turing bands.

Speaking in broad terms, a market sequence is a progressing phase through a series of target prices and tends to show price paths that vary between the straight move up (as in 11111…1) and the phase which is 10101…101 (1 was in the previous example noted as u) knowing that the notation used crunches all consecutive zeros (previously noted H) into a singleton one. This assumption is even stronger when considering large moves are made of larger series of intermediary target prices. Even though not all of the possible *n*-letter chains should be systematically visited within a sequence, at least the different *n*-letter chains should be, on average, visited proportionally to their probabilities of occurrence among the different structural possibilities, as shown in Table 1 [24].

**Figure 4:**
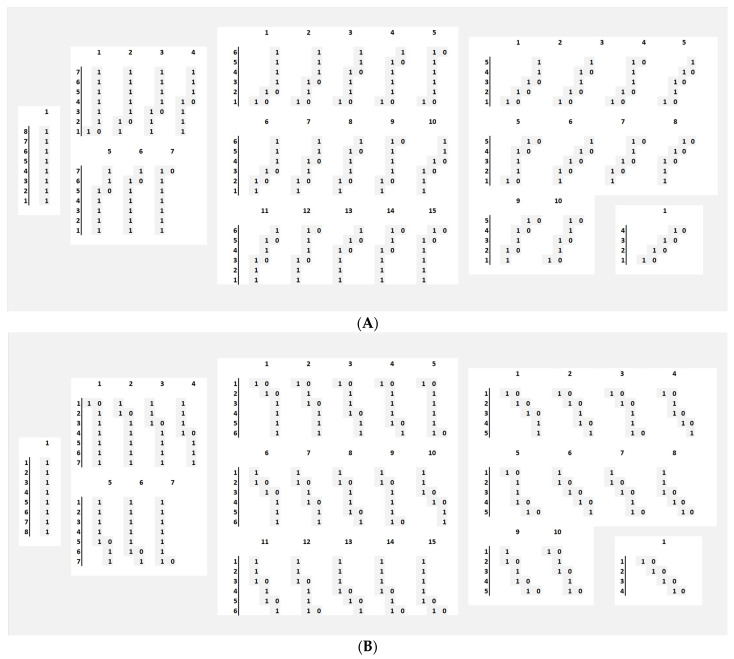
Grouping different ‘tiles’ of an eight-letter chain composed with ‘1’s’ (one tick up or down) and ‘0’s’ (no change). (**A**) shows the set of tiles for moves going up, and (**B**) shows the set of tiles for moves going down.

It is possible to consider the Fibonacci *n*-letter chains expressed in the third column of Table 1 as *tiles*. Each extension represents the length of each class of the different sizes of tiles. For instance, with *n* = 8, the tiles’ set will be as shown in Figure 4. It is noticeable that a tile set covers all the possibilities of a move up or down; in this sense, this representation is mathematically ‘complete’. As shown in Figure 4, with *n* = 8, the *n*-letter chain is made of 34 tiles, composed of 5 different types of tiles of sizes 8, 7, 6, 5 and 4.

**Figure 5:**
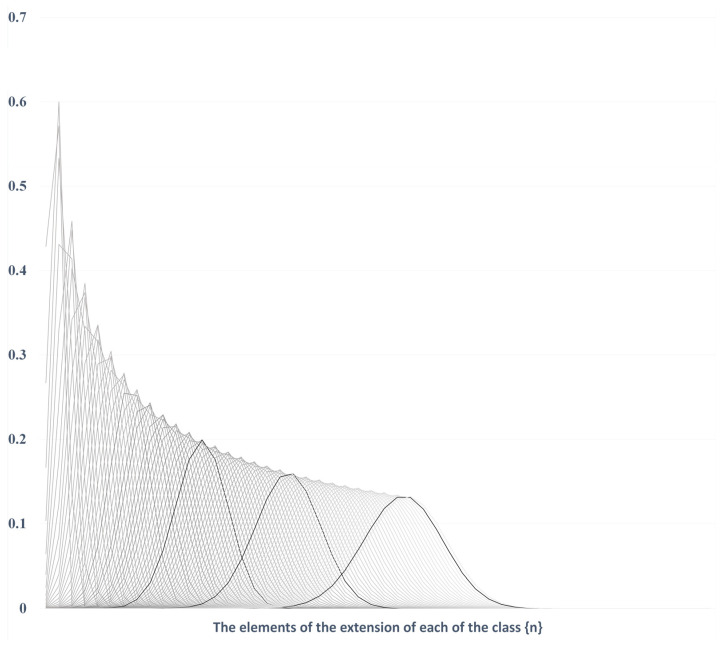
Probability structure of Fibonacci *n*-letter chains, or classes, probability levels on the y-axis.

Given this set of tiles, the probability profile of using these different sets of tiles would be roughly around 3%, 21%, 44%, 29% and 3%, respectively. Taking the 25-price chain, there are 13 sets of tiles instead of 5, and the probabilities of using one tile among the 13 sets are 0.0008%, 0.02%, 0.21%, 1.3%, 4.9%, 12.8%, 22.4%, 26.2%, 20%, 9.4%, 2.5%, 0.30% and 0.01%. With a larger n, the inner structure of the probability profiles is described in Figure 5, where they are put together.

The three different bell curves shown in black in Figure 5 are, from left to right, showing the probabilistic structure of the n-letter chains with *n* = 44, 68 and 100. It shows how the structure evolves when n grows. For the curve of the *n*-letter chain *n* = 100, 99% of the 50 different sorts of tiles will have a length between 79- and 66-unit price, for an average length of 72.4842-unit price (as expressed in footnote 1). For *n* = 44, 99% of the 22 tiles will have a length between 37- and 28-unit price, for an average of around 32.5-unit price. In the previous numerical example, which depicted a move in the euro/dollar, it represents 72.5 and 32.5 pips, (respectively 0.00725 and 0.00325). It is the different tessellations that can be applied to price trajectories that are comparable, without any assumptions regarding the price trajectories themselves.

As expressed in Table 1, the extension E of the *n*-letter chain is for *n* = 8, E{8} = (8 49 90 50 4), i.e., the progress that each of the blocks in Figure 4 describes, and the norm of E{8}, ||E{8}||, 201 units, expresses the sum of all its elements in case all the tiles were used. It is also noticeable that the ratio of two consecutive norms, ||E{*n*+1}||/||E{*n*}|| tends to the golden ratio φ when *n* rises. As such, if considering the n-1 first set of tiles {1},{2},{3} … {*n* − 1}, the sum of their length (norm) will tend to be related to the length of the set of all the tiles the *n*^th^ set contains by a factor of φ when n rises. 

**Figure 6:**
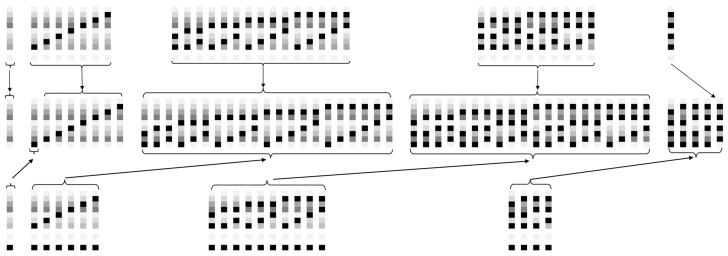
Showing how the tile sets T{9} T{8} T{7} are related. Black squares indicate no price changes (‘H’ or ‘0’), and all other squares indicate a price change of one unit of price (‘1’).

Consecutive tile sets have a structure that has a peculiar spatial behaviour, as shown in Figure 6, where the first line is T{*n* − 1} to which a [1] was added, the middle line is T{*n*}, and the bottom line is T{*n* − 2} to which a tile [1] and a tile [0] were added:

The relationship between tessellation structures is

(10) T {n}=T {n−1} ⋀ [1]+T {n−2} ⋀  [1] ⋀ [0] 

where *T* {*n*} is the set tiles of the class {*n*}, [1] and [0] begin respectively the singleton set with *T* {1} and *T* {0}. Another remarkable relationship links the different blocks within each tessellation class. Considering the function B that takes the set of tiles *T* {*n*} and considers the *p*^th^ block of this set of tiles, *n* and *p* being both integers and *n* being above 3 and *p* above 1,

(11) B(T {n}, p+1)=B(T {n−1} ⋀ [1], p+1)+B(T {n−2} ⋀ [1] ⋀ [0], p) 

As shown in Figure 6, this formula shows that the blocks are translated to the right when n increases. This means that it is always possible to find a better set of tiles to ‘cover’ a move, better being defined as a set of tiles that matches more with the probabilistic profile of each block of the structure by choosing a larger or a smaller set of tiles.

If the ratio of the norms of two consecutive classes tends towards the golden ratio, another remarkable property emerges among the average lengths of each class *n*: they are also related to one another. Given two classes{*n*} and {*p*}, *n* and *p* being integers and such that *n* > *p*, and *p* not among one of the first integers,

(12) $$A(\{n\})\approx A(\{p\})+(n-p)\;*\;\frac{\varphi }{\sqrt{5}}$$

where *A*({*n*}) and *A*({*p*}) are the average length of a price move of the classes {*n*} and {*p*}, and φ is the golden ratio.

(13) $$\frac{\varphi }{\sqrt{5}}=\frac{1}{1+(2-\varphi )}=\frac{1}{1.381966011...}=0.72360679774998\ldots$$

This number, expressed as a continuous fraction, becomes when considering its convergents,

(14) $$\frac{\varphi }{\sqrt{5}}=0+\frac{1}{1+\frac{1}{2+\frac{1}{1+\frac{1}{1+\frac{1}{1+\frac{1}{1+\frac{1}{1+...}}}}}}}=<0;1,2,1,1,1\ldots >$$

Which can be approximated to

(15) $$\sqrt{\frac{\varphi^{2}}{5}}\;\approx \frac{F_{k}}{\varphi^{k}}\approx \frac{F_{k}}{L_{k}}$$

A well-known mathematical result where *F_k_* is the *k*th Fibonacci number, and *L_k_* is the *k*th Lucas number. 

This equation links several noticeable numbers with the differential of *n*-letter class averages. To give numerical examples, and taking two classes, 13 and 31, the average tile lengths are 9.53050398 and 22.554175, respectively, for a difference of 13.0249135, which, multiplied by $\varphi^{18},\;\;i.e.,5777.99983\;gives\;75,257.9482$; at the same time, ${18*F}_{18}=18*4181$ = 75,258. For the sake of comparison, taking two classes, 100 and 7, the average tile lengths are 72.4842 and 5.1904, respectively, for a difference of 69.2938. As $\varphi^{93}=2.72804$ × 10^19^ and $F_{93}=1.97403$ × 10^19^ and n − p = 93, we have, therefore, the left term of the equation A = 69.2938 × 2.72804 ×10^19^ = 1.83585 × 10^21^ and the right term of the equation B = 93 × 1.97403 × 10^19^ = 1.83585 × 10^21^. These numbers are similar down to a factor of 10^−5^.

The physical cutting of a pattern such as the ones shown in Figure 3b, c can be realised using a chosen class{*n*}, and make them n vary in order to find the best fit. The most adequate class{*n*} will be determined by its capacity to provide the set of tiles which provides the best fit to a price sequence, i.e., the best matching class{*n*}—the optimal tessellation—proportionally respecting in the best possible way the probability profile of the Fibonacci *n*-letter chain. There are several factors which help in finding such a class{*n*}: *n* may be the object of a choice, the probability profile of each class allows for approximations, the classes{*n*} are Fibonacci structures and are, as such, efficient tiling structures and, in the end, it is always possible to choose a left composition (Figure 3b) or a right composition (Figure 3c). Finally, what is really at stake is more the fact that identical tiling structures can be applied to different moves: the tiling does not have to be as accurate as it is in the Domino Problem, so the undecidable character of the tiling theory is not an issue.

As in the systemic theory, the relationship, the comparison, literally the ratio between different tiled price sequences is more important than the individual actual tiling of each move. It is the fact that the way they can be the object of a tessellation with structurally identical sets of tiles that matters. This is why the tilling does not have to be optimal; it just has to be as optimal as it can be *in the same manner* within the different sequences or market phases. This manner should be a function of how close price as a measure of value is actually reflecting it, i.e., it can be dictated by human behaviours based on opinions or models -which are ultimately based on opinions- but this manner can even be random or dictated by irrationality.

Without imposing a demanding assumption, it can be said that market participant behaviours, especially considering large moves up and/or down, are relatively constant because they are led by shared market opinions. These opinions are an indeterminable mix of knowledge and ignorance, as stated by Plato. An opinion is an irrational choice; it could be unstable however deemed valid for the period it is believed to be true. It is a mixture of belief and representation and, as such, concerns the future [25]. Shared opinions, more than knowledge itself, are the cement of human societies and are built since consensus is needed to overcome the uncertain future [26,27]. Experts provide a global contextual interpretation from which shared beliefs and opinions of market participants result. Even though they are ultimately based on beliefs, the resulting opinions seem to be closer to conjectures or hypotheses rather than pure beliefs, especially in the financial markets where market participants are well aware of pricing methods and valuation techniques. The most common market proverbs and/or assumptions even reinforce this state of fact: market participants’ decisions could be ‘rational’ and/or could be driven by the couple *fear* and *greed*, as the popular quote says, by another couple, the one opposing right shared opinions or judgments (δόξα), and beliefs or persuasion (πίστις), and/or more theoretically by the mathematical theorem demonstrated by the Goles and Olivos which says that such a dynamical system leads to (a) either a *stabilisation* of opinions or, (b) or an *oscillation* of opinions between two possible configurations C_1_ and C_2_, where the configuration C_1_ leads to C_2_ and C_2_ leads then to C_1_. No other state is possible, i.e., there are no stages of prolonged disorder configurations, three or more states of configuration, and so on [28]. Hence, the way the market behaves along the different market moves is, at least generally, self-similar.

### 3.6. Consequence of Transitivity

The simple fact that it is *most of the time* possible to find a way to replicate any market move within an optimal set of tiles has important consequences. The fact that moves can be described by a structure that is of a self-similar nature is not per se a guarantee that market moves could relate to each other by Fibonacci ratios. Taking into account the Efficient Market Hypothesis (EMH), price changes can only be justified by a change in the news flow: prices are then adjusting, as ‘price reflects value’, at least intrinsic value. Price changes occur because of an adaptation of market participants to new changes. However, the link between this uncertainty and random behaviour is an assumption that has never been proven or demonstrated; the only thing that can be said is that there is a subjective process at work and that the market will slowly adapt to what is believed to be the right price in regards of the perceived value, and (most importantly) considering shared market opinions. The central assumption that supports the use of statistical tools in finance is that a subjective process itself can never lead to identical and self-similar outcomes. 

The way price changes might never be identical, but they follow homomorphic pathways within structures which are related to each other by Fibonacci ratios. Without any normative judgment about these manners, these behaviours, the fact that they, in essence, govern price action in most cases in identical ways is a sufficient condition to legitimate the consideration that the ratio obtained in comparing different market sequences will structurally tend to be the same than the one obtained in comparing the different dimensions of the tiling structure. To summarise the reasoning process:

(i) The way market participants adjust price is said to be motivated by a change in the news flow that affects the matching of value (let us say intrinsic value at least) with price, which are supposed to be equal as most of the time as price reflects value, once more in the context of a set of shared market opinions;
(ii) The dynamic of the voting process reinforces the presence of self-similarity during the progression of market price sequences, irrespective of their dimension [29];
(iii) The reaction to these new informative elements should be self-similar, at least in the long run, as what *causes* the information to permeate into the market price is identical in nature: price does not reflect value anymore (or the opinions and beliefs that most of the market participants have of value);
(iv) Therefore, price should follow similar paths within the self-similar multidimensional structure of tile classes that describe all possible courses of a price trajectory, all the different ways to approach an expected new ‘correct expected price’ or a new target price. This is also true when considering percolation models, described by logistic (Verhulst) functions;
(v) Disregarding how they group in different sets, market participants are, once more, most of the time acting in a similar way when comparing actual prices with their expectations considering the news flow, and this process is mostly a continuous and stable one, here, without even necessitating any demanding qualification regarding the rationality of market participants [3]. It is the process which matters and which is self-similar, even considering the so-called ‘irrational behaviours’ of market participants. Identical configurations of voter expectations generate similar courses among the various tiling classes. In any case, path dependency is a necessary attribute of complex systems [30];
(vi) Hence, the fact that the different dimensions of the tiling structure are related to each other by Fibonacci ratios, be it in absolute terms or in average terms, implies that the effects of price adjustments within market moves motivated by similar causes and are potentially well-described by a self-similar structure will tend to be homeomorphic to this self-similar structure itself;
(vii) Hence, market moves tend to be related to each other by the same ratios as the one found between the different dimensions of the structure;
(viii) Additionally, the greater the price trajectories—the longer they are, the more this will be true, as this tendency will show up even more consistently in larger dimensions. As the process at work is consistent, this structural self-similarity implies fractal characteristics on price trajectories, and the latter brings in an additional constraining factor, multidimensional coherence, which weighs on the way smaller price trajectories are built. Market phases tend to be proportionate to one another, not only between correctly selected increases and decreases but also between moves in similar directions;
(ix) The presence of these ratios is not a necessity. It is rather, and exactly, a structural tendency.

This is coherent, considering:

(i) What value, price and information are one to another, and in particular, the fact they are forming a complex system: price defines value as much as it measures it and is dependent on the information as much as it is part of it;
(ii) That market participants can be largely gathered in more or less consistent groups in the function of their opinions (due to the access to uniformised media, the access to ‘efficient’ information, and the similarity of the valuing tools they use);
(iii) That the structure of each tiling is forgiving in statistical terms, as the very particular profile of the probability structure of Fibonacci *n*-letter chains, as depicted in Figure 5, allows for approximations;
(iv) That the reading of market price histories can be performed using a left or a right composition, which therefore allows greater leeway in the adequation of the actual move and its ‘structured’ profile.

Hence, this structural tendency remains present in all dimensions of significant market phases. Self-similarity drives smaller and larger dimensions to respect these constraints, as path dependency is a necessary attribute of complex systems [30]. A classic hurdle considering all dimensions would be of the same nature as the Sorites paradox regarding sand stacks to such an extent that smaller dimensions reproduce such ratios. The presence of convergent market opinions generates a set of constraints on the way prices are evolving [2]. The presence of shared market opinions destroys the necessity of the causal link between uncertainty and randomness. 

In real-world practices, tools based on Fibonacci ratios are available at every trading station supplied by information and news providers, brokers, trading platforms providers such as Bloomberg, Reuters, eSignal, TradingView, Binance, e-toro and Interactive Brokers because market participants, traders, and fund managers have requested it for decades, independent of the market display chosen (such as Japanese candlesticks, Point and Figure, and Market Profile). On the other hand, theoretical research on this subject is very limited. This study was not intended to provide empirical evidence but aimed to show a theoretical explanation of why these ratios are more subject to appearance than not without stating that they appear at all times (which is, in any case, not true). Future studies may be undertaken to provide an empirical analysis of this model to further back the theoretical explanation.

Regarding limitations, the four-letter alphabet and two subsequent three-letter alphabets used in this study may have shown that a simplistic model could neglect the multifaceted nature of market dynamics. However, financial markets are complex systems, and the simplicity of a model does not affect its capacity to produce or cope with the most sophisticated aspects of the multifaceted nature of market dynamics.

In this study, no consideration of contemporary advancements in stochastic processes and financial theory was given. To our knowledge, such advancements are influenced by an epistemic bias that is linked to the assumptions of the EMH, the hypothesis of randomness and its variations, which are needed to use gas dispersion models to value financial assets [31]. Moreover, the objective of this study was to focus on theoretical discussion and reasoning compared with other literature that either renounces to establish theoretical content on the financial markets and extensively focuses on noise, datasets and empirical substantiation, econophysics, or focuses on behavioural analysis and psychology.

This study may have also assumed that market phases naturally tend to relate to Fibonacci ratios based on self-similarity and homomorphism. However, contrary to Newtonian physics, nature is not neutral in the financial markets, and there is no possible repetition of an experiment (no *ceteris paribus* as such). Despite this, as shown in previous literature, Fibonacci tilings are the most effective tiling systems [32,33,34]. Moreover, percolation models, as stated in this study, provide a theoretical background of why trajectories of these tiling structures have to be in phase with the probability within their *n*-letter classes.

## 4. Conclusions

Fibonacci ratios are reported on the financial markets. This study theoretically explained why these ratios are more subject to appear than not on the markets and thus, why the inherent presence of uncertainty does not causally invalidate forecasting from price history based on them and their derivatives. The presence of shared market opinions has an impact on the interpretation of news and price behaviours and prevents the reduction of indeterminism to randomness. If the main constituents of market activity are stable, the essential similarity of price changes within a set of market expectations could filter the supposed raw impact of news and should tend to translate into Fibonacci proportionate moves, which in turn equally translates into the potential emergence of a structure, as they often appear in complex systems. The implications of this are that the study of both the proportions and the resulting structure could, at least, improve risk management since the fear of collapse is, in essence, identical and independent of the considered asset. This study may bring pragmatic hope for research and a better economic future in which market price behaviours may be better anticipated.

## Figures and Tables

**Table 1:** Pascal’s trade chains and the corresponding Fibonacci *n*-letter chains, extensions and norm.

*n*	Pascal’s Trade Chains	Fibonacci *n*-Letter Chains	Extension (Fibonacci *n*-Letter Chains in Unit Points)	Norm
1	1, 1	1	1	1
2	1, 2, 1	1, 1	2, 1	3
3	1, 3, 3, 1	1, 2	3, 4	7
4	1, 4, 6, 4, 1	1, 3, 1	4, 9, 2	15
5	1, 5, 10, 10, 5, 1	1, 4, 3	5, 16, 9	30
6	1, 6, 15, 20, 15, 6, 1	1, 5, 6, 1	6, 25, 24, 3	58
7	1, 7, 21, 35, 35, 21, 7, 1	1, 6, 10, 4	7, 36, 50, 16	109
8	1, 8, 28, 56, 70, 56, 28, 8, 1	1, 7, 15, 10, 1	8, 49, 90, 50, 4	201
9	1, 9, 36, 84, 126, 126, 84, 36, 9, 1	1, 8, 21, 20, 5	9, 64, 147, 120, 25	365
10	1, 10, 45, 120, 210, 252, 210, …	1, 9, 28, 35, 15, 1	10, 81, 224, 245, 90, 5	655
11	1, 11, 55, 165, 330, 462, 462, …	1, 10, 36, 56, 35, 6	…	…
12	1, 12, 66, 220, 495, 792, 924, …	1, 11, 45, 84, 70, 21, 1	…	…

## Data Availability

The original contributions presented in the study are included in the article, further inquiries can be directed to the corresponding author.
